# Knowledge of caregivers regarding pneumococcal diseases and pneumococcal conjugate vaccine (PCV): A cross sectional study at a district in India

**DOI:** 10.1016/j.jvacx.2024.100532

**Published:** 2024-07-20

**Authors:** Jayanta Majumder, Arindam Ray, Pradeep Haldar, Arup Deb Roy, Debasis Roy, Nitai Chandra Mandal, Tanmay Mahapatra

**Affiliations:** a John Snow India Private Limited, India; b Bill and Melinda Gates Foundation, India; c Ministry of Health and Family Welfare, Government of West Bengal, India; d Piramal Foundation, India

**Keywords:** New vaccine, Pneumococcal diseases, PCV, Caregivers’ knowledge

## Abstract

**Background:**

To reduce burden of pneumonia, India has introduced Pneumococcal Conjugate vaccine (PCV) in routine immunization programme. The state of West Bengal, India introduced PCV in 2021. Uptake of new vaccines depends a lot on knowledge of caregivers on the disease and vaccine**.** This study aimed to assess the knowledge of caregivers regarding pneumococcal diseases and PCV. The study will inform programme managers to develop a comprehensive demand generation strategy for improving uptake of PCV and other new vaccines.

**Methods:**

It is an observational, cross-sectional study using a predesigned, pretested and structured questionnaire conducted among 353 caregivers of children who has received at least one dose of PCV. The children were aged between 6 weeks to 20 months, residing in rural and urban areas of Howrah district of West Bengal. Sample size was calculated considering 95 % confidence interval and 5 % margin of error.

**Results:**

Results are analysed taking into consideration rural/urban divide, socioeconomic status and other factors influencing vaccine uptake. Study findings suggest lack of knowledge of caregivers regarding pneumococcal diseases and PCV. Most of respondents have no idea about any other pneumococcal diseases apart from pneumonia. More than 40 % does not know about what causes pneumonia and more than 47 % does not know how to prevent pneumonia. They also have poor knowledge about injection site, number of doses, schedule and when to start PCV.

**Conclusions:**

Limited knowledge among caregivers may cause negative impact on vaccine coverage and jeopardise the goal of government to reduce morbidity and mortality due to pneumonia.

The study findings suggest that there is dearth of knowledge regarding pneumococcal diseases and PCV among caregivers. Therefore the policy makers need to develop a comprehensive plan for awareness generation for improving PCV uptake and strategy developed for this purpose can be implemented in future new vaccine introduction also.

## Background

Pneumonia is an acute respiratory infection that causes inflammation and accumulation of fluids in the lungs. It makes breathing difficult and limits oxygen intake. Symptoms include fever, chills, cough, rapid and difficult breathing, and chest indrawing [1]. Pneumonia is the leading cause of death among under-5 children, accounting for 15 % of under-5 deaths, globally [2]. In 2018 it caused the death of 808,600 children worldwide, which amounts to 2200 children per day, 92 children per hour and 1 child in every 39 seconds [2]. Children under 2 years of age are more vulnerable to developing severe pneumonia and death. More than 80 % of deaths associated with pneumonia occur in children during the first 2 years of life [4].

Children under 5 years, especially those under 2 years of age are the most at risk of developing and dying from the disease [4]. Besides individuals with compromised immune systems, vulnerable groups such as the elderly, malnourished children, those lacking adequate breastfeeding, individuals exposed to indoor smoke, and those living in crowded conditions, as well as marginalized populations with limited access to healthcare, face a higher risk [3].

Pneumonia can occur due to many bacteria, viruses, and fungi, but the leading cause of bacterial pneumonia under 5 years of age is infection by ***Streptococcus pneumoniae*** [3]**.** It causes pneumococcal pneumonia**.** It accounts for 18 % of all severe pneumonia and 33 % of all pneumonia deaths worldwide [4]. Disease and mortality rates are higher in developing than in industrialized settings, with most deaths occuring in Africa and Asia [5].

Pneumonia continues to kill more children under five worldwide worldwide than any other single infectious disease [6]. Although the problem is worldwide it is most prevalent in developing countries including India [4]. In 2015 in India there were 1.6 million estimated cases of severe pneumococcal pneumonia, accounting for more than 97 % of all severe pneumococcal diseases [4]. In 2015 pneumococcus accounted for 14 % of all under-5 deaths in India [4]. Around 68,700 under 5 deaths were estimated to have occurred in India in 2015 due to pneumococcal diseases [4]. Out of which 53,300 were due to pneumococcal pneumonia [3]. Streptococcus pneumoniae is also known to cause meningitis, sepsis, sinusitis, and infection of the middle ear (Otitis media) [3].

Based on disease burden, safety and efficacy, cost-effectiveness, sustainability, and global evidence, the National Technical Advisory Group on Immunization – India’s apex advisory body on immunization (NTAGI) recommended a phased introduction of Pneumococcal conjugate vaccine or PCV in India’s Universal Immunization Programme (UIP). It was available in the private sector in India before. However, following the pan-India introduction of PCV in 2021, the vaccine is available free of cost for all eligible beneficiaries under the Government’s universal immunization programme. PCV was launched in India in a phase-wise manner from 2017 to 2021. On 29th October 2021, PCV was introduced in West Bengal. PCV is given in a 2 + 1 schedule- two primary doses at the completion of 6 weeks and 14 weeks and a booster dose at 9 months of age. It is expected that with good coverage the burden of pneumococcal diseases and deaths can be reduced.

As in all other health programs, the success of the new vaccine introduction will depend on the awareness level of caregivers. Initiatives have been taken to make people and the overall community to understand the benefits that PCV provides. Lack of awareness can lead to refusal and dropouts leading to poor coverage of the vaccine.

Previous studies in different parts of the world have shown different levels of knowledge regarding pneumococcal diseases and PCV. A 2016 study in Kaduna state of Nigeria [7] showed that 77.2 % of respondents were aware of pneumonia but 68 % had poor knowledge of pneumonia. A 2014 study [8] illustrated that knowledge about pneumococcal diseases and willingness to vaccinate their children with PCV is much higher in the already vaccinated group than the unvaccinated group. Another 2016 study In Shanhai [9] shows that 61.9 % knew about pneumonia and 68.1 % had heard about the vaccine.

A North Delhi study in 2019–2020 [10] found that only 58.5 % of females and 41.9 % of males have heard about pneumococcal infection and 66.5 % of respondents said that PCV is necessary for newborns.

A recent study (2021–22) at Medical College in Kolkata [11] just after the PCV launch showed only 30.7 % of parents knew about pneumococcal diseases and 31.7 % had any knowledge about PCV. PCV is a recently launched vaccine in India.

Though the PCV introduction started in 2017 in a majority of the states it started in 2021 in the middle of the COVID pandemic. There have been few studies to assess the knowledge and awareness level of caregivers about the disease and the vaccine post-pan-India introduction in India. This study plans to capture the awareness and knowledge level of caregivers regarding pneumococcal diseases and PCV.

### 1.1 Aim

To assess the knowledge and awareness level of caregivers regarding pneumococcal diseases and PCV.

### 1.2 Objectives

To identify the knowledge & awareness level of caregivers regarding pneumococcal diseases and PCV that can be used to plan measures to be taken to improve awareness if found necessary by the study.

### 1.3 Methodology

The present study is an observational cross-sectional study.

## 2 Study design

The present study was conducted in West Bengal, a state of India located in the eastern region of the country. Population-wise it is the fourth largest state in India but area-wise it is only the thirteenth largest in the country. It was the last of the 36 states and union territories to launch PCV on 29th October 2021. Two rural blocks and one urban area of the Howrah district were selected for the study for ease of transport as the investigators needed to visit the areas every week for almost 3 months. The blocks included in the study were Rural − Panchla, Domjur, and urban −Uluberia municipality. Data collection was done in the month of May, June, July, and August of 2023.

Using the formula,

$$\text{SS}\;=\left[{\text{Z}}^{2}\text{p}\;\left(1-\;\text{p}\right)\right]/{\;\text{C}}^{2}$$

SS = Sample size; Z = Given Z value; p = Percentage of population; C = Confidence level

A sample of 333 was calculated. The sample size was calculated considering a 95 % confidence interval and 5 % margin of error using the following formula.

Confidencelevel=95%

Marginoferror=±5%

Population proportion having any knowledge about pneumococcal conjugate vaccine (PCV) = 31.7 % [11]

Where P1 = 31.7 %, Q1 = 100-P1; Z = 1.96; D = 5.

Sample size: 333

Only caregivers coming to immunization session sites with children aged between 6 weeks to 20 months who have received at least one dose of PCV were included in the study. The included caregivers were all mothers. In a few cases, the father/grandmother/aunt accompanied the mother, but it is the mother who provided consent and answered the questions.

The Six-week to 20-month age group was selected keeping in mind the schedule of the first dose of PCV was at the completion of 6 weeks, the launch of the vaccine in West Bengal was on 29th October 2021 and data collection started in May 2023.

Session sites of all 4 urban primary health centers were utilized for data collection at Uluberia municipality. However, in blocks, there were many session sites each week, so they were chosen randomly.

The final sample size included in the study was 353. Out of the included participants, 198 were from 2 rural blocks (102 from Panchla and 96 from Domjur) and 155 participants were from Uluberia municipality.

### 2.1 Study tools

The tool for data collection was a set of pre-designed and pre-tested structured questionnaire. Apart from basic demographic information it comprised of 2 sections – Knowledge of pneumococcal disease and knowledge about PCV. The questionnaire was translated into the local language Bengali.

### 2.2 Ethical consideration

The study was approved by the ethics committee of Barrackpore Population Health Research Foundation. Permission was also taken from the Department of Health and Family Welfare, Government of West Bengal After explaining the study, informed consent (translated into the local Bengali language) was taken from all respondents for their voluntary participation.

### 2.3 Data collection and analysis

Caregivers coming with their children at session sites for immunization were explained about the nature and purpose of the study. Only after their consent, the questionnaire was administered to them. First basic demographic information and socioeconomic status were recorded. Then questionnaire on pneumococcal diseases were administered followed by questions on PCV. Urban and rural data were collated separately so that any significant difference could be easily identified. Then all data were added together for data analysis. Data analysis was done using Microsoft Excel and SPSS 25.

### 2.4 Results

Results were analyzed taking into consideration the rural/urban divide, socioeconomic status, and other factors influencing vaccine uptake.

### 2.5 Demographic and Socio-economic profile

Out of 353 children whose mothers were interviewed 182 (51.6 %) were male and the rest were female. 181(51.3 %) of them were first born and 36.3 % were second child of their parents. Out of 353 mothers, 81.3 % were between 18 to 30 years. Rest was above 30 years of age. Only 4 (1.1 %) were illiterate. 8.8 % were educated up to the primary level only, and 26.9 % were at middle school level. 27.8 % were educated up to high school level, 24.1 % were of intermediate level, 7.1 % were graduate and 4.2 % were post graduate.

93.8 % of mothers were homemakers and the rest were working. 18 of the 22 working mothers were jari workers (a typical local style of dressmaking) and 4 of them were teachers. The majority of respondents were Muslims (59.2 %) and the rest were Hindus. Only 17.3 % of them were of general caste. The rest were either Other backward classes (OBCs) or Scheduled castes (SCs). Both are considered as socioeconomically backward classes.

A majority (67.1 %) of the families of respondents consisted of 3–5 members. Financial status was calculated based on the modified Kuppuswamy scale of 2022. Families with a monthly income of less than INR 9307 were 38.5 %. Families in the next level with monthly income up to INR 27882 were 51 %. The next two higher levels consisted of only 9.4 % and 1.1 % only. In Table 1 monthly income is converted to annual income by multiplying it by 12.

**Table 1:** Demographic and socio-economic characteristics of respondents (n = 353).

**Characteristics**	**Categories**	**Number**	**Percentage**
**Gender of Child**			
	Male	182	51.6
	Female	171	48.4

**Order of Birth**			
	1	181	51.3
	2	129	36.5
	3	32	9.1
	4	11	3.1

**Age of Mother/Caregiver**			
	18–30 years	287	81.3
	> 30 years	66	18.7

**Education Qualification of Mother**			
	Illiterate	4	1.1
	Primary school	31	8.8
	Middle school	95	26.9
	High school	98	27.8
	Intermediate/Diploma	85	24.1
	Graduate	25	7.1
	Postgraduate/Professional	15	4.2

**Occupation**			
	Unemployed/Homemaker	331	93.8
	Other profession	22	6.2

**Religion**			
	Hindu	144	40.8
	Muslim	209	59.2

**Caste**			
	General	61	17.3
	OBC	209	59.2
	SC	83	23.5

**Family size**			
	≤5	237	67.1
	>5	116	32.9

**Annual Family Income (Rs.)**			
	<111,684	136	38.5
	111,696–334,584	180	51
	334,596–557,688	33	9.4
	557,700–834,408	4	1.1

### 2.6 Caregivers’ knowledge, perception, and practice about pneumococcal diseases

The study revealed that 92.9 % of respondents had “heard about pneumococcal diseases”. However, when asked to name a few pneumococcal diseases only 11 out of 353 respondents were able to come up with more than one name. Out of 328 respondents, 327 of them said pneumonia is a pneumococcal disease. Only one of the respondents opined that it is a type of cough and cold. Out of these 327 people who named pneumonia as pneumococcal disease, 9 of them also named meningitis, and 2 of them named sinusitis as pneumococcal disease. No one talked about otitis media.

Regarding symptoms of pneumonia, 12.2 % of respondents had no idea, 56.7 % said patients suffer from fever, 6.5 % said patients may have chills, 77.6 % said cough is one of the symptoms, 28.3 % said breathlessness, 5.7 % talked about rapid respiration and 4.8 % talked about in drawing of chest wall.

When it comes to the causes of pneumonia, 40.2 % had no idea. Only 33.4 % said it occurs due to some infection, while 32 % blamed exposure to cold weather for pneumonia. In total, only 7 people talked about malnutrition, indoor smoke, and overcrowding as possible causes and 3 respondents blamed mosquito bites for pneumonia. Low immunity and lack of breastfeeding were not mentioned by anyone.

When asked about the susceptible age group 64.9 % said under-2 years children, 41.1 % mentioned under-5, and 9.1 % mentioned old age. 11.3 % thought that it may happen at any age and 19.8 % did not know the age group (see Table 2).

**Table 2:** Caregivers’ knowledge, perception, and practice regarding pneumococcal diseases (n = 353).

**Variables**	**Options**	**Number**	**Percentage**
**Heard about Pneumococcal diseases**			
	Yes	328	92.9
	No	25	7.1

**If yes, name a few Pneumococcal diseases (n = 328) (Multiple responses allowed)**			
	Pneumonia	327	99.7
	Meningitis	9	2.7
	Sinusitis	2	0.6
	Otitis Media	0	0
	Others (Cough and Cold)	1	0.3

**Signs and Symptoms of Pneumonia (Multiple responses allowed)**			
	Fever	200	56.7
	Chill	23	6.5
	Cough	274	77.6
	Rapid Respiration	20	5.7
	Breathlessness	100	28.3
	Indrawing of Chest Wall	17	4.8
	Don't know	43	12.2

**Causes of Pneumonia (Multiple responses allowed)**			
	Infection	118	33.4
	Low Immunity	0	0
	Malnutrition	3	0.8
	Lack of Breast Feeding	0	0
	Indoor Smoke	1	0.3
	Overcrowding	3	0.8
	Exposure to Cold	113	32
	Others (Mosquito Bite)	3	0.8
	Don't know	142	40.2

**Most Susceptible Age Group (Multiple responses allowed)**			
	Under 2 yr.	229	64.9
	Under 5 yr.	145	41.1
	Old age	32	9.1
	Any age	40	11.3
	Don't know	70	19.8

**Can Pneumococcal Diseases Cause Hospitalization**			
	Yes	295	83.6
	No	17	4.8
	Don't know	41	11.6

**Can Pneumococcal Diseases Cause Death**			
	Yes	235	66.6
	No	44	12.5
	Don't know	74	20.9

**How to Prevent Pneumonia (Multiple responses allowed)**			
	Exclusive Breast Feeding	6	1.7
	Improving Nutrition	4	1.1
	Vaccination	168	47.6
	Avoiding Indoor Smoke and Overcrowding	6	1.7
	Other (avoiding cold weather)	12	3.4
	Don't know	167	47.3

**Has the Child Ever Suffered From Pneumonia or Meningitis Since Birth**			
	Yes	24	6.8
	No	325	92.1
	Don't know	4	1.1

**If yes, Who is Contacted First for Treatment (n = 24)**			
	ASHA	1	4.2
	Private Doctor	15	62.5
	Government Hospital	8	33.3

When asked about if the pneumococcal disease may require hospitalization, 83.6 % answered in the affirmative, 4.8 % said it was not required and 11.6 % did not know. When asked if pneumococcal disease may cause death, 66.6 % agreed, 12.5 % were of the opinion that it never causes death, and 20.9 % had no knowledge about it.

Respondents were asked about how they think pneumonia can be prevented and 47.3 % had no knowledge regarding this, 3.4 % were of the view that avoiding cold weather will prevent pneumonia.

Exclusive breastfeeding, improving nutrition, avoidance of smoke, and overcrowding totaled only 4.5 %. On the other hand, 47.6 % said vaccination can prevent pneumonia.

The respondents were asked whether their child had ever suffered from pneumonia or meningitis since birth. 24 or 6.8 % answered in the affirmative. Then questions were asked about their health-seeking behavior. Only 1 out of 24 parents went to Accredited Social Health Activist (ASHA, Community mobilizer for immunization programme) first. 62.5 % (n = 15) went to private doctors and 33.3 % (n = 8) of them went to government hospitals (see Table 2).

### 2.7 Caregivers’ knowledge and attitude about PCV

Among the respondents, 300 (85 %) believe that PCV works, 51 (14.4 %) said they did not know whether PCV works or not, and only 2 (0.6 %) of them said that PCV is not effective. It is a matter of concern that even after their children were provided with one or more doses of PCV, 15 % of respondents are still not sure whether it works or thinks it does not work.

Out of 300 respondents, 299 said that PCV prevents pneumonia and one said it prevents cough and cold. None of the respondents mentioned meningitis, sinusitis, or otitis media.

When asked how PCV is given, 81.6 % said PCV is injectable, 15.6 % had no idea how PCV is provided and 2.8 % said it is given orally.

Among the respondents, 58.1 % were able to mention correctly that the injection was to be given to the right thigh. 25.5 % had no idea, 8.5 % said left thigh, 6.2 % said right arm, and left arm was mentioned by 1.7 %.

Only half of the respondents (50.4 %) said that 3 doses of PCV were required, and 34.5 % did not know the number of doses required. The rest of them provided wrong answers like 1, 2, >3 doses (See Table 3).

**Table 3:** Caregivers’ knowledge and attitude about PCV (n = 353).

**Variables**	**Options**	**Number**	**Percentage**	**p value (Chi-square test)**
**Does PCV work**				
	Yes	300	85	0.00*
	No	2	0.6	
	Don't know	51	14.4	

**If yes, then diseases against which PCV gives protection from (n = 300)**				
	Pneumonia	299	99.7	0.00*
	Meningitis	0	0	
	Sinusitis	0	0	
	Otitis Media	0	0	
	Others (Cough and Cold)	1	0.3	

**Route of Administration**				
	Oral	10	2.8	0.00*
	Injection	288	81.6	
	Don't know	55	15.6	

**Site of Administration**				
	Right Arm	22	6.2	0.00*
	Left Arm	6	1.7	
	Right Thigh	205	58.1	
	Left Thigh	30	8.5	
	Don't know	90	25.5	

**No. of Required Doses of PCV**				
	1	26	7.4	0.00*
	2	26	7.4	
	3	178	50.4	
	>3	1	0.3	
	Don't know	122	34.5	

**When to Start PCV**				
	6 Weeks	188	53.2	0.00*
	10 weeks	7	2	
	14 weeks	7	2	
	Other	11	3.1	
	Don't know	140	39.7	

**Doses schedule (Appropriate timing of all 3 PCV doses)**				
	Correct	109	30.9	0.00*
	Incorrect	77	21.8	
	Don't know	167	47.3	

**No. of PCV Doses received**				
	1	136	38.5	0.02*
	2	96	27.2	
	3	121	34.3	

**Any Previous Dose/s Taken from Private Facility**				
	Yes	1	0.3	
	No	352	99.7	

**If Yes, How Many (n = 1)**		1		
**Is PCV Safe**				
	Yes	321	90.9	0.00*
	No	0	0	
	Don't know	32	9.1	

**Are you willing to provide your child with PCV if it is not given free**				
	Yes	175	49.6	0.01*
	No	178	50.4	

**Would you be able to provide your child with PCV if it is not given free**				
	Yes	125	35.4	0.00*
	No	228	64.6	

**Source of Information Regarding PCV**				
	ASHA	331	93.8	0.00*
	ANM	21	5.9	
	Other HW (FTS)	1	0.3	

* p < 0.05 considered as statistically significant.

**Fig. 1:**
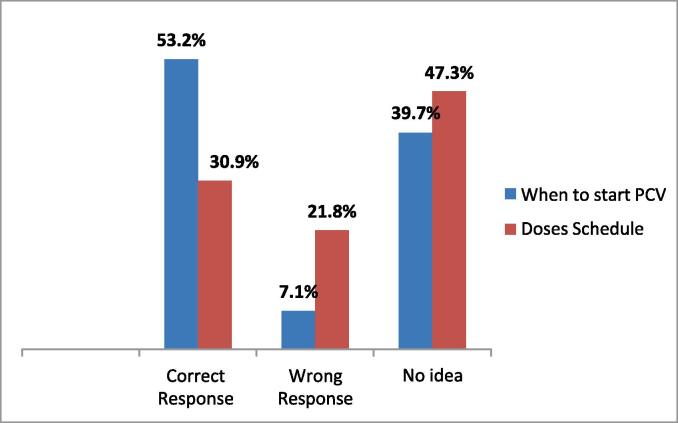
Caregivers knowledge on PCV.

Respondents were also asked about when to start PCV. The correct answer of 6 weeks was given by 53.2 % of them, 39.7 % had no idea and the rest provided wrong answers (see Fig. 1).

Only 30.9 % of respondents had correct knowledge of the PCV schedule, 21.8 % provided the wrong answer and almost half of them (47.3 %) did not know the schedule.

On being asked about the number of doses that had been provided to their children, 38.5 % of respondents said their child had taken 1 dose, 27.2 % had taken 2 doses and the rest had taken all 3 doses. Out of 353 children, only 1 had taken PCV from a private clinic.

90.9 % of respondents believe that PCV is safe, rest did not know. Nobody said PCV is unsafe.

Among the respondents, 49.6 % said they are willing to provide PCV to their children if it is not given free, but only 35.4 % said that they will be able to bear the cost of it. 93.8 % of respondents received PCV-related information from ASHA, 5.9 % from Auxiliary Nurse and Midwife (ANM – vaccinator in immunization programme) and only 0.3 % received information from First Tier Supervisor (FTS − vaccinator when ANM is not available).

### 2.8 Rural-urban divide

As mentioned earlier 2 rural blocks and one urban area in Howrah district were chosen for data collection. 198 respondents were from the rural areas of Panchla and Domjur, and 155 respondents were from Uluberia Municipality. Geographically, Panchla is in the middle. It is connected with both Uluberia and Domjur. Though Uluberia is an urban area, it is surrounded by rural blocks and socio-economically not much ahead of adjacent blocks. All 3 areas are Muslim-majority areas. In our study also the picture was same regarding education, family size, occupation of mothers, religion, and caste except in urban area 16.1 % of families are in an annual income range of Rs.331776 and above, whereas in rural areas only 6.1 % are in the same income bracket.

**Fig. 2:**
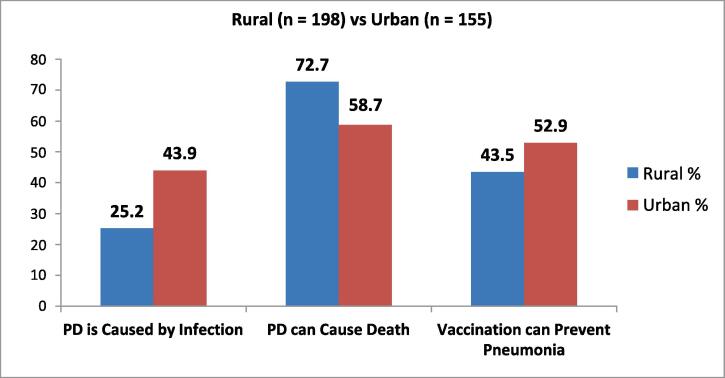
Caregivers’ Knowledge regarding Pneumococcal diseases (Rural vs Urban).

Regarding knowledge about pneumococcal diseases, in rural areas, only 25.2 % think it is due to infection, whereas in the urban area, 43.9 % think infection is the main reason. In rural areas, 72.7 % of respondents said that pneumococcal diseases can cause death, but the percentage drops down to only 58.7 % in urban area. But in urban area, 52.9 % of respondents believe vaccination can prevent pneumonia, but in rural areas, only 43.5 % think so (See Fig. 2). 9 % of respondents in urban area complained that their child has suffered from pneumonia, but in rural areas it is only 5.1 %. After pneumonia more than 70 % of caregivers in urban area took their child to a private doctor, this practice is only 50 % in rural areas.

**Fig. 3:**
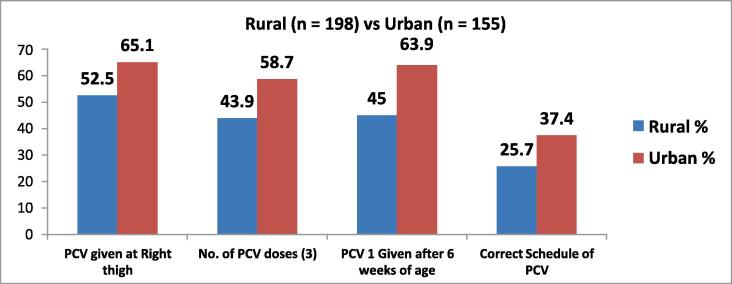
Caregivers’ Knowledge regarding PCV (Rural vs Urban).

Overall knowledge about PCV is found to be more in urban area. 65.2 % of urban respondents knew that PCV injection is given at the right thigh, this number is much lower (52.5 %) in rural areas. Only 43.9 % rural caregivers knew that PCV had 3 doses, but 58.7 % of urban respondents knew about it. In urban area, 63.9 % knew that PCV 1st dose was to be given after 6 weeks of age, but only 45 % of caregivers in rural areas had the knowledge. Also, only 25.7 % of rural respondents knew the correct schedule of PCV, this knowledge was higher (37.4 %) among urban respondents (See Fig. 3).

If PCV is to be taken in private then only 43.9 % in rural areas are willing to provide it to their children, but due to high prices, only 26.3 % will be able to provide it. In urban area both these numbers are higher (56.8 % and 47.1 %) respectively.

**Fig. 4:**
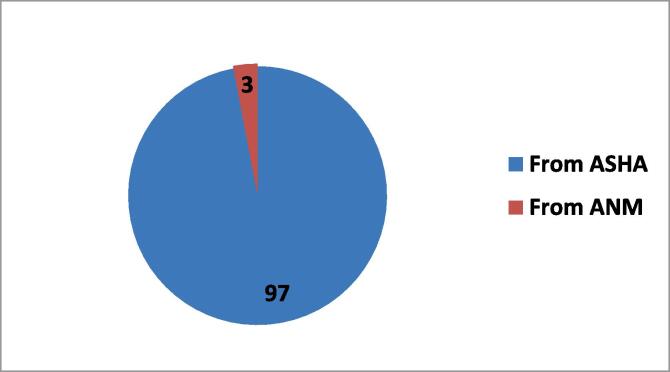
Provider of PCV-related Information in Rural Areas.

**Fig. 5:**
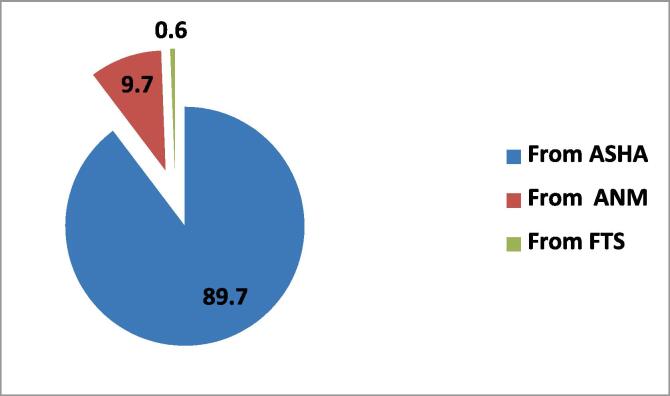
Provider of PCV-related Information in Urban Area.

In rural areas, 97 % of respondents get their PCV-related information from ASHA, compared to 89.7 % in urban area. ANMs are also a significant (9.7 %) source of information in urban area (See Fig. 4, Fig. 5)

## 3 Discussion

The study was conducted in 2 rural blocks (Panchla and Domjur) and 1 urban area (Uluberia Municipality) of Howrah district. Most (93.8 %) of them were housewives. 328 out of 353 respondents (92.9 %) said that they have heard about Pneumococcal diseases. Out of that 328 respondents, 327 of them said they had heard about pneumonia, but when questioned further only 11 of them were able to mention the name of any other pneumococcal disease. Our findings were in line with a study conducted in Kaduna state of Nigeria [7] which reported that 77.2 % of respondents were aware of pneumonia but 68 % had poor knowledge of pneumonia.

The majority of the respondents knew about symptoms like fever and cough. However, they had poor knowledge about breathlessness, rapid respiration, indrawing of chest wall, chill.

Regarding pneumonia-related hospitalization and death, 83.6 % were aware that pneumonia may need hospitalization and 66.6 % were aware that severe pneumonia may even cause death. This was in contrast with a study conducted in Kolkata [11] which reported that only 10.1 % had knowledge about the fatality of pneumococcal diseases. This difference in findings could be because, at the time of the study [11], PCV had been recently launched. However, our study was conducted almost 2 years post-launch of PCV allowing people to be better informed about pneumococcal diseases and PCV.

Only 47.6 % considered vaccination as a way of preventing pneumonia and (47.3 %) do not know how pneumonia can be prevented. Regarding knowledge about the vaccine, 85 % (n = 300) believed that PCV actually works, and barring one respondent, all of them said that it prevents pneumonia. The respondents did not mention any other disease which is potentially prevented by PCV.

These findings were in line with a study conducted in North Delhi [10] where 66.5 % of respondents said that PCV is necessary for newborns.

Among the caregivers, 81.6 % knew that PCV is given as an injection and 58.1 % had correct knowledge about the site of injection. 50.4 % of respondents knew correctly that PCV is given 3 times under the UIP schedule. Furthermore, 53.2 % knew the correct age of administration of PCV first dose. Another area of concern is that only 30.9 % had correct knowledge about the schedule.

Though the majority of them took their child to a private doctor when they had pneumonia, when it came to vaccination with PCV, almost all of them (352 out of 353) took it from a government facility. Though many of them lack basic knowledge about PCV, more than 90 % of respondents think PCV is safe and mostly get vaccine-related information from ASHA. It shows caregivers’ faith in UIP and frontline health workers.

Among the respondents, 49.6 % said they are willing to provide PCV to their children even if it is not given free, but only 35.4 % said that they will be able to bear the cost of it. This percentage is quite low compared to the Singapore study [8] where 94.4 % of respondents from the vaccinated group were willing to pay for the vaccine if needed. This may be due to the difference in financial status of the two study areas.

Overall, to most of the caregivers, pneumococcal disease is pneumonia and PCV protects from pneumonia only. They hardly know about other diseases that come under pneumococcal diseases and that can be prevented by PCV. Compared to another study in Kolkata [11] conducted just after the PCV launch in the state, the knowledge level among caregivers is better in this study. But there is plenty of scope for improvement.

## 4 Conclusion

In conclusion, our study sheds light on a critical gap in caregiver knowledge concerning pneumococcal diseases and the Pneumococcal Conjugate vaccine (PCV) in the state of West Bengal, India. The findings underscore a significant lack of awareness among caregivers, in both rural and urban areas, and across various socioeconomic strata. This knowledge deficit extends to fundamental aspects such as the causes of pneumonia, prevention measures, and key details about the PCV, including injection site, dosing, schedule, and initiation age.

The implications of this study are far-reaching, emphasizing the pressing need for targeted and comprehensive educational initiatives. Program managers can leverage these insights to design and implement effective demand-generation strategies aimed at enhancing PCV uptake and, by extension, improving overall immunization coverage. Bridging the informational gap among caregivers is crucial not only for the success of the PCV program but also for laying the groundwork for the acceptance of future vaccines.

As we strive to reduce the burden of pneumonia, it is imperative to recognize the role of caregiver knowledge as a cornerstone of successful immunization programs. Addressing these knowledge gaps will not only empower caregivers to make informed decisions about their children's health but will also contribute to the broader public health goal of preventing pneumococcal diseases. Future research and interventions should focus on tailoring educational efforts to specific demographic groups, considering the unique challenges faced by caregivers in different settings. Through collaborative efforts between healthcare providers, policymakers, and communities, we can pave the way for a more informed and resilient healthcare landscape, ultimately leading to improved child health outcomes.

## CRediT authorship contribution statement

**Jayanta Majumder:** Writing – review & editing, Writing – original draft, Visualization, Validation, Software, Methodology, Investigation, Formal analysis, Data curation, Conceptualization. **Arindam Ray:** Writing – review & editing, Supervision, Software, Resources, Project administration, Funding acquisition, Data curation, Conceptualization. **Pradeep Haldar:** Writing – review & editing, Visualization, Supervision, Resources, Project administration, Conceptualization. **Arup Deb Roy:** Writing – review & editing, Writing – original draft, Visualization, Supervision, Software, Resources, Project administration, Methodology, Investigation, Funding acquisition, Formal analysis, Conceptualization. **Debasis Roy:** Writing – review & editing, Visualization, Validation, Supervision, Resources, Investigation, Formal analysis, Conceptualization. **Nitai Chandra Mandal:** Writing – review & editing, Visualization, Validation, Supervision, Resources, Investigation, Conceptualization. **Tanmay Mahapatra:** Writing – review & editing, Visualization, Software, Methodology, Investigation, Formal analysis, Data curation, Conceptualization.

## Declaration of competing interest

The authors declare that they have no known competing financial interests or personal relationships that could have appeared to influence the work reported in this paper.

## Data Availability

Data will be made available on request.
